# Trehalose Enhances Long-term Recovery in 18-month-old Mice by Increasing Autophagy after Traumatic Brain Injury

**DOI:** 10.1093/geroni/igab046.3538

**Published:** 2021-12-17

**Authors:** Rodney Ritzel, Yun Li, Jordan Carter, Niaz Khan, Junyun He, Samantha Allen, Alan Faden, Junfang Wu

**Affiliations:** University of Maryland School of Medicine, Baltimore, Maryland, United States

## Abstract

Older patients with traumatic brain injury (TBI) have higher mortality and poorer long-term outlook compared to younger individuals. This may contribute to the assumption that aggressive management of geriatric TBI is futile. The present study examined the long-term recovery potential and underlying mechanisms associated with advanced age in male C57BL/6 mice using a controlled cortical impact model of TBI. Older (18 mos) mice had higher mortality compared to younger (10 wks) mice at 12 weeks post-injury. While aging alone had a profound impact on behavioral ability, the recovery slope in some, but not all, neurobehavioral tests was relatively similar between young and old injured mice. NanoString analysis identified several age- and injury-specific genes that were differentially expressed, including those involved with the complement, phagocytosis, and autophagy pathways. Flow cytometry demonstrated dysregulation of autophagic function in microglia with normal aging which was exacerbated after TBI. Given the critical role for autophagy in promoting the cellular degradation of cytoplasmic materials, we reasoned that treatment with the autophagic inducer, trehalose, may be a viable therapeutic strategy. Trehalose was administered in the drinking water (3%) starting at d1 post-injury up to 8 weeks. Older TBI mice treated with trehalose exhibited either delayed deficits or enhanced recovery in cognitive and motor tasks. Trehalose modified expression of autophagy markers and reprogrammed the microglial response to TBI. Our data indicate that microglia undergo chronic changes in autophagic regulation that are associated with poor outcome. Boosting autophagy may be a promising therapeutic strategy for older TBI patients.

